# Correlation of Chest X-Ray Scores in SARS-CoV-2 Patients With the Clinical Severity Classification and the Quick COVID-19 Severity Index

**DOI:** 10.7759/cureus.24864

**Published:** 2022-05-09

**Authors:** Vo Tan Duc, Tran Thi Mai Thuy, Nguyen Hoang Nam, Ha Thi Bich Tram, Truong Thi Phuong Thao, Lam Thuy Doan, Le Nguyen Gia Hy, Le Nguyen Diem Quynh, Nguyen Hong Duc, Le Minh Thang, Le Duy Mai Huyen, Phan Cong Chien, Le Huu Hanh Nhi, Uyen Do, Le Huu Nhat Minh

**Affiliations:** 1 Department of Diagnostic Imaging, University Medical Center, Ho Chi Minh City, VNM; 2 Department of Radiology, University of Medicine and Pharmacy at Ho Chi Minh City, Ho Chi Minh City, VNM; 3 Department of Radiology, Vinmec Healthcare System, Ho Chi Minh City, VNM; 4 Nelda C. Stark School of Nursing, Texas Woman’s University, Houston, USA; 5 Faculty of Medicine, University of Medicine and Pharmacy at Ho Chi Minh City, Ho Chi Minh City, VNM

**Keywords:** chest x-ray (cxr), covid-19, chest x-ray, rale score, covid-19 severity, brixia score

## Abstract

Objectives

This study aimed to assess the role of chest X-ray (CXR) scoring methods and their correlations with the clinical severity categories and the Quick COVID-19 Severity Index (qCSI).

Methods

We conducted a retrospective study of 159 COVID-19 patients who were diagnosed and treated at the University Medical Center between July and September 2021. Chest X-ray findings were evaluated, and severity scores were calculated using the modified CXR (mCXR), Radiographic Assessment of Lung Edema (RALE), and Brixia scoring systems. The three scores were then compared to the clinical severity categories and the qCSI using Spearman's correlation coefficient.

Results

Overall, 159 patients (63 males and 96 females) (mean age: 58.3 ± 15.7 years) were included. The correlation coefficients between the mCXR score and the Brixia and RALE scores were 0.9438 and 0.9450, respectively. The correlation coefficient between the RALE and Brixia scores was marginally higher, at 0.9625. The correlation coefficients between the qCSI and the Brixia, RALE, and mCXR scores were 0.7298, 0.7408, and 0.7156, respectively. The significant difference in the mean values of the three CXR scores between asymptomatic, mild, moderate, severe, and critical groups was also noted.

Conclusions

There were strong correlations between the three CXR scores and the clinical severity classification and the qCSI.

## Introduction

There have been more than 200 million confirmed cases of COVID-19 worldwide, with over four million associated deaths [1]. The manifestations of this illness are variable, from asymptomatic to significant respiratory failure situations or even deaths [2]. According to the World Health Organization (WHO), the severity of the disease is classified into six groups: asymptomatic infection, mild pneumonia, severe pneumonia, acute respiratory distress syndrome (ARDS), sepsis, and septic shock [3].

In cases diagnosed with pneumonia, evaluation of disease severity based solely on clinical judgment may result in ambiguous decisions. Severity scores have been advocated as valuable tools to assist physicians with outcome prediction and decision-making. The Brescia-COVID Respiratory Severity Scale (BCRSS) and the Quick COVID-19 Severity Index (qCSI) are two clinical risk prediction scores that are of special interest [4,5]. The qCSI was obtained from documents of COVID-19 patients admitted to hospitals in the northwestern United States. It was developed with the main aim of predicting critical respiratory failure within 24 hours [6]. Before beginning any medical therapy for patients with COVID-19, evaluation of the seriousness of the disease by a chest X-ray (CXR) performed at hospital admission should be considered. Imaging examination is widely claimed to play a pivotal role in COVID-19 management [7-9]. Currently, there are some established CXR scoring systems for quantifying lung abnormalities, including the Brixia and Radiographic Assessment of Lung Edema (RALE) classifications. The Brixia score has been used to evaluate the severity and monitor the course of COVID-19 pneumonia in China and Italy, while the RALE scoring system has been applied in some hospitals in Shenzhen [10]. The required experience of radiologists is one of the primary distinctions between the two scoring systems. The RALE score is designed to be simpler than the Brixia score so that a general practitioner can use it easily [11]. However, the RALE scoring system is not explicit to smaller lesions because it divides the lung into just two regions [12,13]. Recently, a modified scoring system, developed from the Brixia and the RALE, which was attempted to apply to cases of mild pneumonia and provide a more convenient diagnostic tool, was being applied in an Indonesian hospital. The results of the study in this hospital showed that the modified CXR (mCXR) score had a close correlation with clinical severity [13]. The aim of this study was to determine the role of CXR scoring systems in assessing the severity of COVID-19 in correlation with the clinical severity classification and the qCSI.

## Materials and methods

This is a retrospective study of patients with COVID-19 confirmed by reverse transcription-polymerase chain reaction (RT-PCR) at the University Medical Center, Ho Chi Minh City, Vietnam. The protocol was reviewed and approved by the Human Research Ethics Committee of the University Medical Center, Ho Chi Minh City.

Study settings and subjects

We collected data of 159 patients who were admitted to the emergency department from July 2021 to August 2021 and had both RT-PCR-confirmed COVID-19 and CXR examinations. The inclusion criteria were as follows: COVID-19-positive patients who presented at the University Medical Center, Ho Chi Minh City, and underwent CXR scanning. The exclusion criteria were as follows: patients suspected of COVID-19 infection yet refused to take confirmatory tests.

The initial CXRs at admission were analyzed by two groups of radiologists with experience of four years and over four years. If there was no consensus between the two groups, we discussed and consulted with the radiologist who had an experience of over 10 years to obtain the ultimate results.

Clinical data

We recorded the age, sex, and comorbidities of the 159 patients. The recorded clinical factors included SpO_2_, respiratory rate (RR), and patient oxygen requirement at admission.

Regarding clinical severity classification, the patients were divided into five categories: asymptomatic infection, mild disease, moderate pneumonia, severe pneumonia, and critical situation. The asymptomatic group was composed of patients having no clinical symptoms. The mild group included patients having acute upper respiratory tract infections such as fever, dry cough, sore throat, nasal congestion, exhaustion, headache, muscle aches, and tongue numbness and no signs of pneumonia or hypoxia, with respiratory rate (RR) ≤ 20 breaths/minute and SpO_2_ ≥ 96% on room air. The moderate pneumonia group had signs of pneumonia such as fever, cough, dyspnea, fast breathing over 20 breaths/minute, and SpO_2_ ≥ 93% on room air. The severe pneumonia group had a fever or suspected respiratory infection accompanied by any of the following: RR over 30 breaths/minute, severe dyspnea, or SpO_2_ < 93% on room air. The critical group consisted of acute respiratory distress syndrome (ARDS), sepsis, or septic shock. Sepsis was diagnosed when any of the following signs of organ dysfunction were present: alterations of consciousness (drowsiness, lethargy, and coma), respiratory abnormalities (dyspnea, fast breathing, and low oxygen saturation), circulatory abnormalities (tachycardia, weak pulse, cold extremities, hypotension, and skin mottling), renal dysfunction (oliguria or anuria), laboratory evidence of coagulation abnormalities, thrombocytopenia, acidosis, high lactate, and hyperbilirubinemia. Septic shock was defined as the presence of any of the following conditions: persistent low blood pressure in spite of volume resuscitation, requiring vasopressors to keep mean arterial blood pressure (MAP) no less than 65 mmHg, and serum lactate level above 2 mmol/L.

Quick COVID-19 Severity Index

The Quick COVID-19 Severity Index (qCSI) was calculated using three variables that can be readily evaluated at the bedside. Respiratory rates ≤ 22, from 23 to 28, and over 28 breaths/minute were given points 0, 1, and 2, respectively. The points of oxygen flow rates under 2, 3-4, and over 5 L/minute were 0, 4, and 5, respectively. SpO_2_ values above 92%, from 89% to 92%, and ≤88% have 0, 2, and 5 points, respectively. The total score was then used to divide patients into four groups: 0-3, low risk; 4-6, low-intermediate risk; 7-9, high-intermediate risk; and 10-12, high risk.

Image analysis

All chest radiographs were acquired in posteroanterior or anteroposterior projection. The radiologists then categorized the radiographs as normal or abnormal. CXR alterations were represented according to the types of lesions (reticular opacities, nodular opacities, ground-glass opacities (GGO), or consolidations), side (right lung, left lung, or bilateral), craniocaudal distribution (upper zone, middle zone, lower zone, or diffuse), and peripheral dominance. If an opacity was on the outer third of the hemithorax, it was deemed peripheral. The presence of additional features, such as pleural effusion or nodules, was also recorded. The typical pattern was regarded as ground-glass opacity or consolidation having the characteristic bilateral and peripheral distribution in the mid to low zone of the lungs. Other than the aforementioned pleuropulmonary abnormalities were atypical.

Brixia scale

In the Brixia scoring system, the lungs were divided into six regions by two lines. The first line was located at the level of the inferior wall of the arch of the aorta, and the second line was at the level of the right inferior pulmonary vein. Each region was assigned a score ranging from 0 to 3 based on the severity of lung damage. Normal CXR, interstitial infiltration, combined type infiltration with interstitial predominance, and combined type infiltration with alveolar predominance were given 0, 1, 2, and 3 points, respectively. The total scores of the six lung zones varied from 0 to 18. Other abnormalities, such as pleural effusion and enlargement of pulmonary vessels, were excluded from this scoring system.

RALE scale

In the RALE score, each lung was assigned a score of 0-4 depending on the extent of involvement by consolidation or alveolar opacities: 0 points for no participation, 1 point for <25% participation, 2 points for 25%-50% participation, 3 points for 50%-75% participation, and 4 points for >75% participation. The maximum score for this scoring system was 8.

Modified chest X-ray scoring system

**Figure 1:**
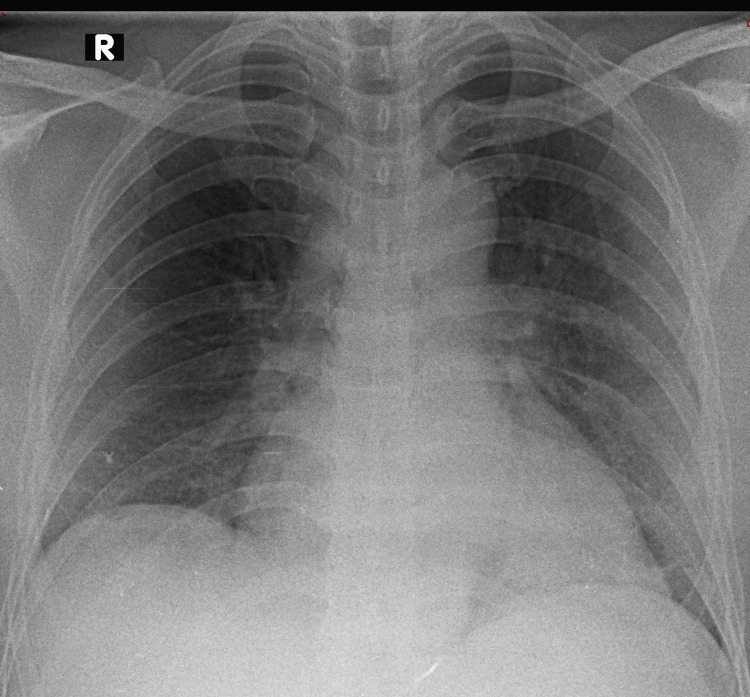
Typical radiographic pattern in a moderate SARS-CoV-2 pneumonia case This CXR showed reticular, ground-glass opacities and consolidations in bilateral lower and mid-zones. In the bilateral mid-zones, the lesion had a peripheral predominant distribution. The Brixia score was 9, the RALE score was 5, and the mCXR score was 7. This case was classified as moderate pneumonia based on clinical severity. The qCSI was 4.

**Figure 2:**
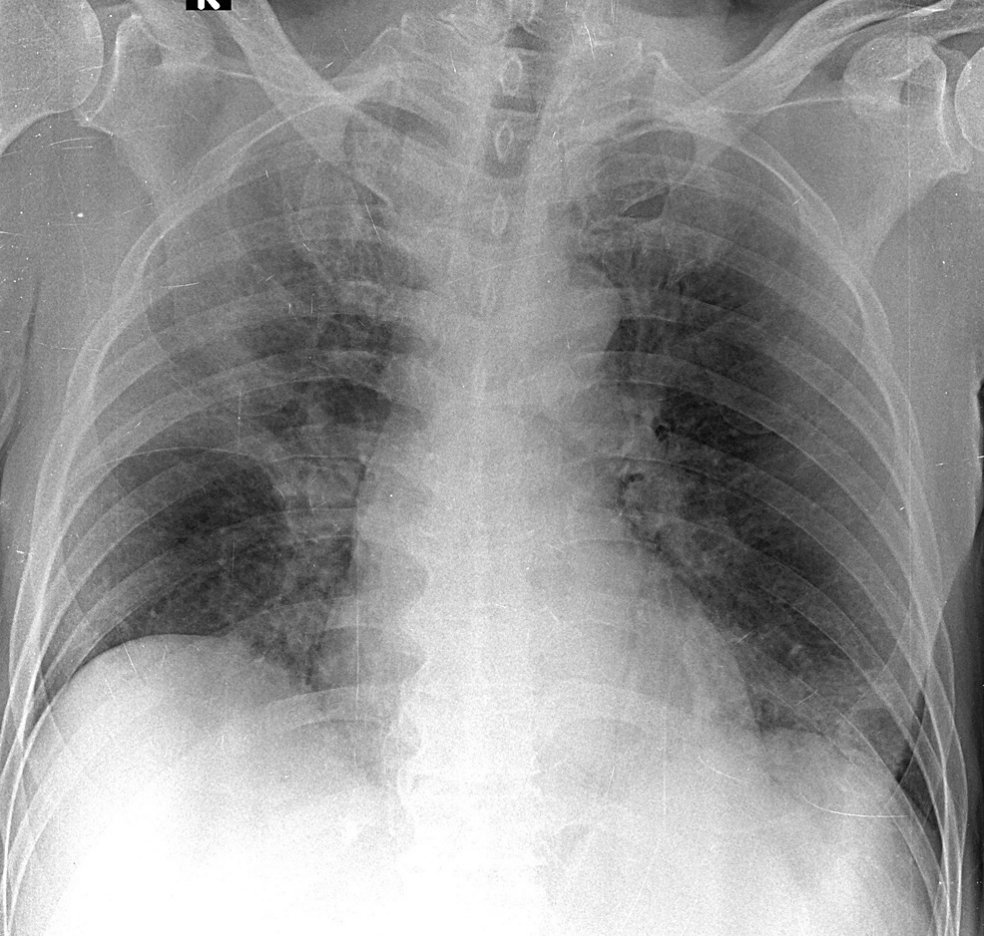
Atypical radiographic pattern in a moderate SARS-CoV-2 pneumonia case This CXR showed ground-glass opacity in the right peripheral lower zone and consolidations in the right upper lobe and left basal zone. The Brixia score was 12, the RALE score was 4, and the mCXR score was 8. This case was classified as moderate pneumonia based on clinical severity. The qCSI was 2.

**Figure 3:**
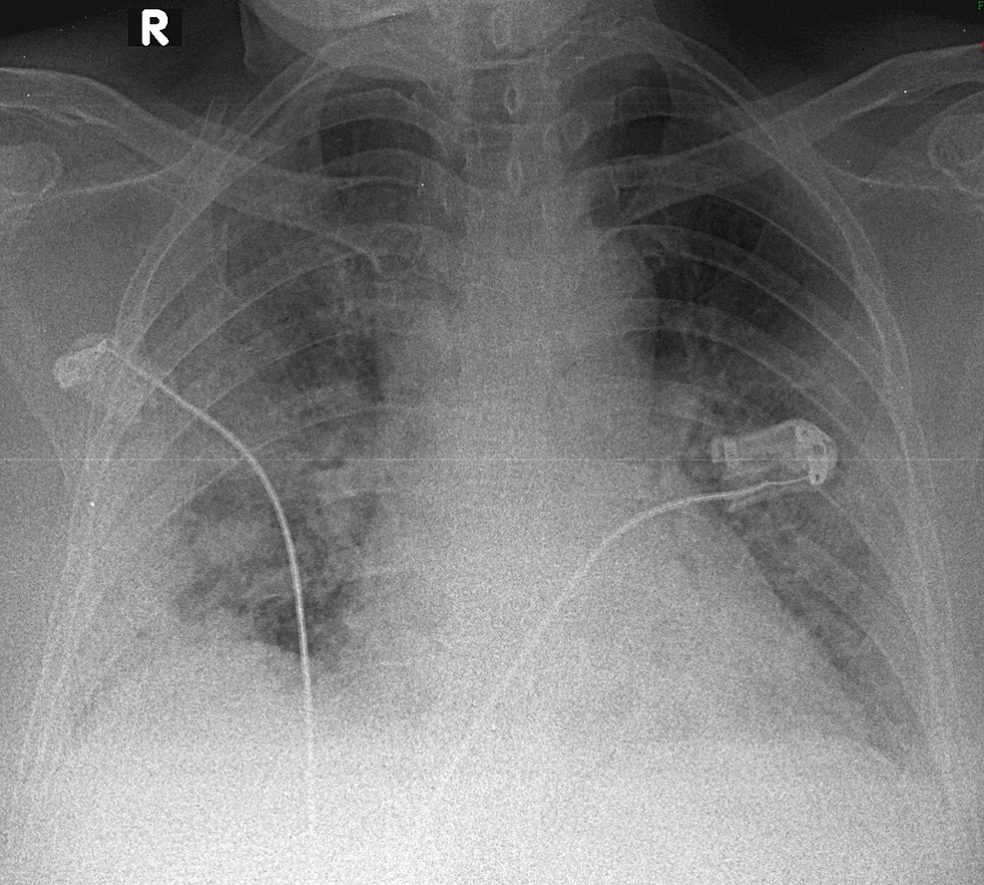
Typical radiographic pattern in a critical case of SARS-CoV-2 pneumonia This CXR showed ground-glass opacities and consolidations in bilateral, peripheral lower, and mid-zones. The Brixia score was 12, the RALE score was 7, and the mCXR score was 9. This case was classified as a critical situation based on clinical severity. The qCSI was 9.

**Figure 4:**
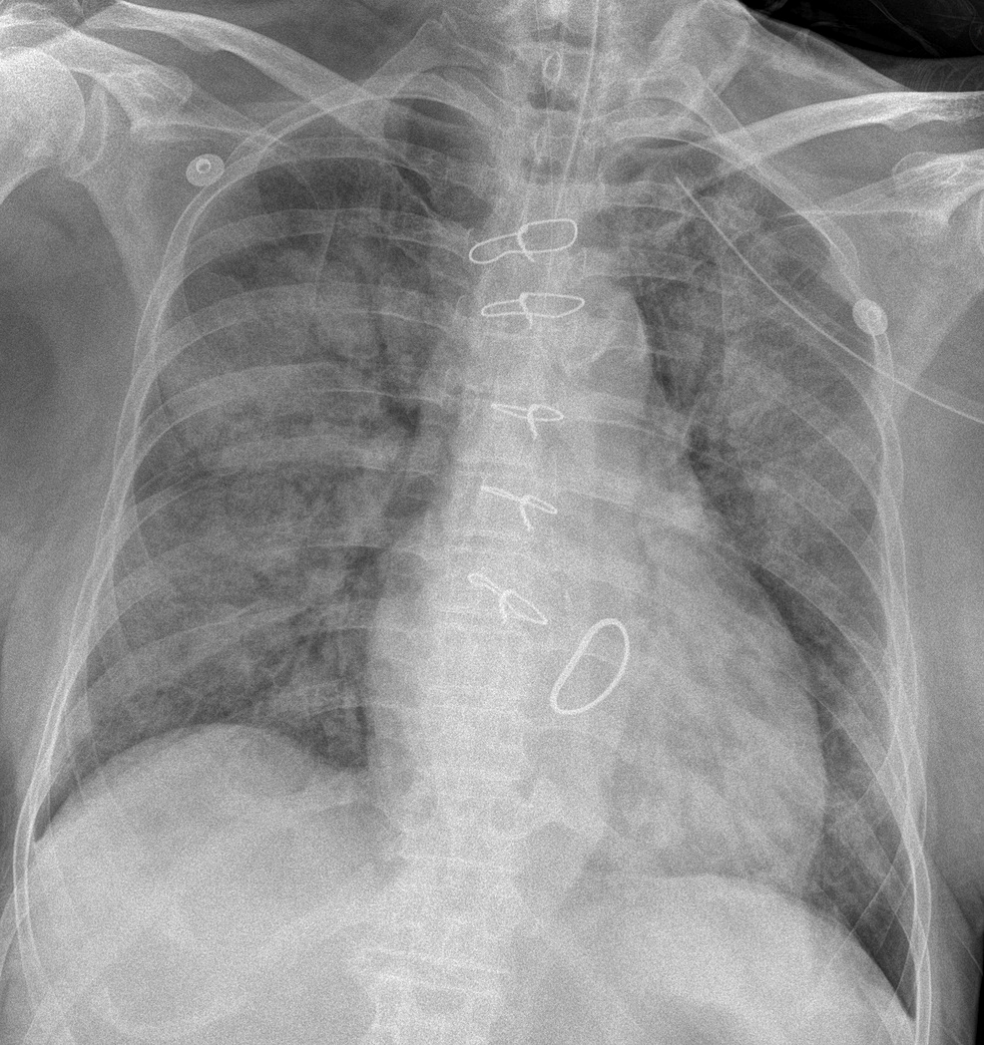
Atypical radiographic pattern in a critical SARS-CoV-2 pneumonia case The CXR showed diffuse consolidations in bilateral lung fields with the air bronchogram sign. The Brixia score was 18, the RALE score was 8, and the mCXR score was 12. This case was classified as a critical situation based on clinical severity. The qCSI was 10.

In the modified chest X-ray scoring system (mCXRSS), two horizontal lines were used to divide the lungs into six fields. Each field was scored from 0 to 2 according to radiological findings: 0 if no abnormalities were noted, 1 if infiltrates or consolidations were less than 50%, and 2 if infiltrates or consolidations were more than 50%. The maximum score for this grading system was 12. The total score was then classified further into three following categories: mild (1-4), moderate (5-8) (Figures 1, 2), and severe (9-12) (Figures 3, 4).

We analyzed the data using STATA 14.0 (STATA Corp., Texas, USA). Data are shown as frequency and percentage for qualitative variables and mean values ± SDs or median and interquartile range for quantitative variables. The Shapiro-Wilk test was used to assess the normality of the distribution of each scoring system. Spearman's correlation test was used to measure the degree of association between the mCXRSS with the RALE and Brixia scoring scale and with clinical classification. The Mann-Whitney U test and Kruskal-Wallis rank test were used for two or more independent samples. Differences were considered statistically significant if p-values were under 0.05 in all analyses.

## Results

Patient characteristics

The patient characteristics of this study are presented in Table 1. The current study included 63 men (39.6%) and 96 women (60.4%), with a mean age of 58.3 ± 15.7 (21-91) years. Patients predominantly presented with severe pneumonia (38%) and had comorbidities (80.5%). Hypertension was the most common comorbidity (42.8%). The three most common symptoms on admission were fever, cough, and dyspnea. Regarding the qCSI, most cases had a score of 0 to 3, indicating a low risk of pulmonary failure within 24 hours after admission. The mean SpO_2_ was 89.8% ± 9.9%.

**Table 1:** Sample characteristic

Parameter	Number of patients (%) (n = 159)
Patient characteristic	
Male	63 (39.6%)
Female	96 (60.4%)
Comorbidities	
No comorbidities	31 (19.5%)
Hypertension	68 (42.8%)
Gastritis	55 (34.6%)
Diabetes	45 (28.3%)
Heart abnormalities	30 (18.9%)
Hepatitis	20 (12.6%)
Lipid disorder	13 (8.2%)
Chronic renal failure	13 (8.2%)
Stroke	13 (8.2%)
Cancer	4 (2.5%)
Obesity	4 (2.5%)
Pregnancy	3 (1.9%)
Symptoms on admission	
Fever	104 (65.8%)
Cough	93 (58.5%)
Dyspnea	91 (57.2%)
Loss of smell	3 (1.9%)
Loss of taste	4 (2.5%)
Clinical feature classification	
Asymptomatic	17 (10.7%)
Mild pneumonia	33 (20.9%)
Moderate pneumonia	34 (21.4%)
Severe pneumonia	60 (38%)
Critical situation	15 (9.4%)
Risk classification based on the qCSI	
Low	85 (53.5%)
Low-intermediate	16 (10.1%)
High-intermediate	20 (12.6%)
High	38 (23.9%)
Clinical index	Mean (SD)
qCSI	4.4 (4.6)
SpO_2_	89.8 (9.9)

Chest radiography features

Out of 159 patients, 47 (19.6%) had normal CXRs and 112 (70.4%) had CXR abnormalities (Table 2). In 112 abnormal CXRs, all cases presented with reticular opacities, ground-glass opacities (GGO), and consolidations. Most of the cases (41.6%) had a combination of two pulmonary lesion types. The distribution of lesions was mostly two or three zones (32.1% and 40.2%, respectively). Of the 112 patients with CXR abnormalities, 103 (92%) presented with bilateral lesions, 28 (25%) were peripheral dominant, and 33 (29.5%) had middle and lower distribution. Of the cases, 15.7% had the typical pattern of COVID-19 pneumonia, which was previously defined as ground-glass opacity or consolidation having the bilateral, peripheral, and mid to lower lung zone distribution. The mean value of the Brixia, RALE, and mCXR scores were 6.9, 3.5, and 5.2, respectively.

**Table 2:** Radiographic findings on chest radiographs

Radiographic characteristic	Number of findings (%) (n = 159)
Normal	47 (29.6%)
Typical pattern	25 (15.7%)
Atypical pattern	87 (54.7%)
Radiographic characteristic	Number of findings (%) (n = 112)
One pattern of lesion	38 (33.9%)
Reticular opacities	17 (15.2%)
Ground-glass opacities (GGO)	17 (15.2%)
Consolidation	4 (3.6%)
Combination of two patterns	66 (58.9%)
Reticular opacities + GGO	23 (20.5%)
Reticular opacities + consolidation	10 (8.9%)
GGO + consolidation	33 (29.5%)
Combination of three patterns	8 (7.1%)
Distribution of lesion	
One zone	31 (27.7%)
Upper	1 (0.9%)
Middle	3 (2.7%)
Lower	27 (24.1%)
Two zones	36 (32.1%)
Upper and middle	1 (0.9%)
Upper and lower	2 (1.8%)
Middle and lower	33 (29.5%)
Three zones (upper, middle, and lower)	45 (40.2%)
Unilateral	9 (8%)
Right lung	5 (4.5%)
Left lung	4 (3.6%)
Bilateral	103 (92%)
Peripheral predominant	28 (25%)
No predominant distribution	84 (75%)
Chest X-ray score	Mean (SD)
Brixia	6.9 (5.7)
RALE	3.5 (2.8)
mCXR	5.2 (4)

Chest radiography scoring and clinical features

There were no statistically significant differences between the mean values of the Brixia, RALE, and mCXR scores in the gender group and in the comorbidity group. The patients were divided into three groups according to age: under 30 years, 30-50 years, and over 50 years. There were no statistically significant differences between the mean values of the Brixia, RALE, and mCXR scores in these three groups.

The statistically significant difference in the mean values of the three chest X-ray scores between low-risk, low-intermediate-risk, high-intermediate-risk, and high-risk groups classified according to the qCSI is shown in Table 3.

**Table 3:** Chest X-ray scores in risk classification based on the qCSI

	Low risk	Low-intermediate risk	High-intermediate risk	High risk	p-value
Brixia	3.5 ± 4.7	8.4 ± 4.6	10.5 ± 3.7	12.0 ± 3.7	0.0001
RALE	1.7 ± 2.2	4.3 ± 2.3	5.2 ± 1.8	6.2 ± 1.7	0.0001
mCXR	2.8 ± 3.4	6.6 ± 3.3	7.5 ± 2.3	8.8 ± 2.4	0.0001

The statistically significant differences in the three CXR scores between asymptomatic, mild, moderate, severe, and critical groups classified according to clinical classification are recorded in Table 4.

**Table 4:** Values of the three scoring systems in five categories of clinical severity

	Asymptomatic	Mild disease	Moderate pneumonia	Severe pneumonia	Critical situation (ARDS, sepsis, and septic shock)	p-value
Brixia	0.9 ± 2.0	1.9 ± 3.4	6.5 ± 4.7	10.0 ± 4.3	13.0 ± 3.9	0.0001
RALE	0.5 ± 1.1	0.9 ± 1.6	3.2 ± 2.3	5.0 ± 2.2	6.6 ± 1.8	0.0001
mCXR	0.7 ± 1.5	1.8 ± 3.0	4.9 ± 3.4	7.4 ± 2.9	9.2 ± 2.7	0.0001

According to Table 5, the majority of patients were classified as mild according to the mCXR score (65 patients, 40.9%). In the mild mCXR score group, most patients were asymptomatic or presented with mild disease (43/65 patients, 66.2%). Those classified as moderate by the mCXR score mostly had moderate or severe pneumonia at presentation (42/52 patients, 80.8%), while those deemed as severe by the mCXRSS mostly had severe pneumonia or critical situations (35/42 patients, 83.3%).

**Table 5:** mCXR severity score in five categories of clinical severity

	Asymptomatic	Mild disease	Moderate pneumonia	Severe pneumonia	Critical situation (ARDS, sepsis, and septic shock)	Total	Percentage
Mild	16	27	14	7	1	65	40.90%
Moderate	1	5	14	28	4	52	32.70%
Severe	0	1	6	25	10	42	26.40%
Total	17	33	34	60	15	159	100%

Spearman's correlation coefficient between the mCXR score and the Brixia score was 0.9438 (p < 0.001), and that between the mCXR score and the RALE score was 0.9450 (p < 0.001). Besides, Spearman's correlation coefficient between the RALE score and the Brixia score was slightly higher, which was 0.9625 (p < 0.001). This showed that the RALE score had marginally higher correlation coefficients relative to both the Brixia and mCXR scoring systems (Table 6).

**Table 6:** Spearman's correlation coefficients between the mCXR, Brixia, and RALE scores

	Brixia score	Modified chest X-ray (mCXR) score
RALE score	r = 0.9625	r = 0.9450
	p < 0.001	p < 0.001
	n = 159	n = 159
mCXR score	r = 0.9438	
	p < 0.001	
	n = 159	

There was an inverse correlation between SpO_2_ and the Brixia score, RALE score, and mCXR score with Spearman's correlation coefficients of -0.6740, -0.6949, and -0.6701, respectively. The correlation coefficient between the qCSI and the Brixia score was 0.7298 (p < 0.01) and between the qCSI and the RALE score was 0.7408 (p < 0.001). The correlation coefficient of the qCSI and the mCXR score was 0.7156 (p < 0.001). This indicated that the RALE score had a slightly higher correlation coefficient relative to both SpO_2_ and qCSI (Table 7).

**Table 7:** Spearman's correlation coefficients between the three chest imaging scoring systems and SpO2 and qCSI

	SpO_2_	qCSI
Brixia score	r = -0.6740	r = 0.7298
	p	p < 0.001
	n = 159	n = 159
RALE score	r = -0.6949	r = 0.7408
	p < 0.001	p < 0.001
	n = 159	n = 159
Modified chest X-ray (mCXR) score	r = -0.6701	r = 0.7156
	p < 0.001	p < 0.001
	n = 159	n = 159

## Discussion

The clinical features of COVID-19 patients manifest a broad spectrum of severity [2]. The Vietnamese clinical grading system of severity classified COVID-19 patients into five categories: asymptomatic patient, mild disease, moderate pneumonia, severe pneumonia, and critical situation [3,14]. In this study, asymptomatic patients made up 10.7% of the total, mild patients made up 20.9%, moderate patients made up 21.4%, severe patients made up 38%, and critical patients made up 9.4%. Within 24 hours of admission, a significant number of COVID-19 patients have respiratory failure. According to much research, the Quick COVID-19 Severity Index (qCSI) could be a simple and accurate method to predict the risk of severe respiratory failure within 24 hours [5]. The qCSI, ranging from 0 to 12 points, measures respiratory rate, nasal cannula flow rate, and minimum documented pulse oximetry being available at the bedside. The risk of decompensation in the group with qCSI of 0-3 was only 4%, whereas those of patients with a score of 4-6, 7-9, and 10-12 were 30%, 44%, and 57%, respectively [5]. In our study, most patients (53.5%) had a low risk of respiratory failure within 24 hours of hospitalization, and the percentages of low-intermediate, high-intermediate, and high risk of decompensation were 10.1%, 12.6%, and 23.9%, respectively.

According to Cleverley et al., COVID-19 patients may exhibit clinical symptoms or radiographic changes of pneumonia late despite normal initial chest radiographs. A case series analysis of 64 hospitalized COVID-19 patients reported that 31% had normal chest radiographs at admission [15]. In our study, 29.6% of all patients had normal chest radiography at admission, but we did not examine the follow-up images, so this may be studied in further research. Progression of COVID-19 infection may cause dense areas on CXR known as ground-glass opacities, which are defined as increased density in the lung parenchyma with the pulmonary vessels still being visible [16]. Consolidation has a similar appearance to ground-glass opacity, except for being denser and making pulmonary vessels invisible [3]. Lung nodules and lymphadenopathy are rarely seen [3]. In this study, no cases with pleural effusions, lung nodules, or lymphadenopathy were recorded. According to a previous study, a characteristic chest radiographic pattern could correctly identify COVID-19 with a sensitivity of 15.5%, specificity of 96.6%, positive predictive value of 83.8%, and negative predictive value of 50.1% [17]. Rousan et al. stated that peripheral ground-glass opacities affecting the lower lobes were the most common findings on CXR [18]. In this study, abnormal CXR findings included reticular opacities, ground-glass opacities, and consolidations. Most of the cases (66%) had a combination of two or three pulmonary lesion types. Involvement was usually two or three zones (32.1% and 40.2%, respectively). CXR abnormalities had a predominantly peripheral distribution (25%) and middle and lower zone distribution (29.5%) with bilateral involvement (92.0%). Of the patients, 15.7% had the typical pattern on chest X-rays. The mechanism of bilateral peripheral distribution could be explained as SARS-CoV-2 predominantly attacks type II pneumocytes located in the peripheral and subpleural areas of the lungs. These cells then undergo apoptosis, which causes a release of viral substances to adjacent pneumocytes, propagating the disease [9]. COVID-19 pneumonia has a classic manifestation on chest radiography. It may appear as patchy or extensive geographical consolidation. The lesions are often bilaterally, peripherally, and basally located. Consolidation occurs when alveolar air spaces are filled with inflammatory exudate and edema. It leads to a dense projection on CXR in a relatively characteristic distribution. However, the disease can have widespread lung involvement in several patients, displaying a nonspecific morphology [9]. In this study, up to 54.7% of cases had atypical patterns on CXR. Recognizing both common and nonspecific imaging appearances of COVID-19 pneumonia is pivotal to improving patient care. Although a diagnosis of COVID-19 pneumonia should not be made just based on a chest radiograph, it can be an important part of the diagnostic pathway, along with RT-PCR results.

In the study of Rousan et al., 783 patients with COVID-19, including 532 males and 251 females, were enrolled [18]. Men had significantly higher Brixia scores than women only in the age group of 50-79 years. In both genders, there was a strong association between the Brixia score and age [19]. According to the work of Monaco et al., a median Brixia score of 8 was observed, without a significant difference between males and females (p = 0.758). However, this study showed a significant yet weak correlation between the Brixia score and age (ρ = 0.177, p = 0.002) [20]. Our study included 63 men (39.6%) and 96 women (60.4%), with a mean age of 58.3 ± 15.7 (21-91) years. We did not find any statistically significant differences in the Brixia, RALE, and mCXR scores between the two genders. The patients were divided into three age groups: <30 years, 30-50 years, and >50 years. There were no significant differences between the values of the Brixia, RALE, and mCXR scores of these three groups.

Patients who presented with severe clinical scenarios in the study of Setiawati et al. scored 6 or above with the Brixia system [13]. In the research conducted by Kim et al., 410 CXRs were evaluated using the RALE system. In that study, blood oxygen saturation and CXR grade were both significantly associated with the length of hospital stay. Moreover, oxygen saturation and RALE score were significant predictive factors of intubation [21]. The study of Setiawati et al. reported that most patients clinically graded as "severe" scored 5 or more with the RALE system [13]. Setiawati et al. stated that although the Brixia score was helpful for risk-stratifying COVID-19 patients, this scoring system required trained radiologists because of its complexity. The RALE score can predict the need for supplemental oxygen, ICU admission, and mechanical ventilation, yet it lacks detailed and dedicated descriptors [22]. Therefore, doctors of an Indonesian hospital suggested the use of the modified chest X-ray (mCXR) scoring system [13]. The mCXR score was considered convenient, but there was not much research on this. Therefore, in our study, besides the Brixia score and the RALE score, we utilized the mCXR score and found that the majority of patients were classified as mild based on this system (65 patients, 40.9%). In the mild mCXR score group, most patients presented with asymptomatic or mild disease (43/65 patients, 66.2%). Those classified as moderate on CXR mostly had moderate and severe pneumonia (42/52 patients, 80.8%), while those classified as severe by the mCXR score mostly had severe pneumonia and critical situations (35/42 patients, 83.3%).

Many researchers proved that the qCSI was valuable in predicting ICU admission [5,6]. However, there were no reports about the relations between CXR scores and the qCSI in previous studies. Our study showed a statistically significant difference in the mean values of the three chest X-ray scores between the low-risk, low-intermediate-risk, high-intermediate-risk, and high-risk groups classified according to the qCSI. The higher the CXR score was, the higher the qCSI the patients obtain. This study is a premise for the following research with a larger sample size to illustrate the role of qCSI and CXR scores in the initial evaluation of COVID-19 patients.

The study of Setiawati et al. showed that the three scoring systems correlated well with one another. Particularly, the mCXR scoring system correlated more toward Brixia (p < 0.01; correlation coefficient: 0.865) than RALE (p < 0.01; correlation coefficient: 0.855). This indicated the reliability of the mCXR scoring system in assessing the severity of COVID-19 pneumonia, which helps determine early management. Nevertheless, we must be aware of the fact that COVID-19 patients do not always present with pneumonia [13]. The mCXR scoring system assists healthcare workers to evaluate COVID-19 pneumonia simply and speedily. Our study showed that the mCXR score was highly correlated with both the Brixia and RALE scores, with correlation coefficients of 0.9438 (p < 0.01) and 0.9450 (p < 0.01), respectively. The correlation coefficient between Brixia and RALE scores was marginally higher, which was 0.9625 (p < 0.01). The RALE score had higher correlation coefficients relative to both Brixia and mCXR scores.

The current study was not without limitations. Firstly, the sample size was rather small. Secondly, imaging data were recorded based on the initial CXR at hospital admission. Further studies on the efficacy of the scoring systems on a larger and more heterogeneous population should be conducted because of the usefulness of their implementation at healthcare centers that lack CT scanners. The investigation of a correlation between the scoring of serial radiographs and patient outcomes will greatly contribute to patient prognostication and monitoring of disease progression.

## Conclusions

In the fight against COVID-19, imaging modalities are gaining worldwide attention because of their usefulness and approachability. In particular, chest radiography can be used to assess the severity of COVID-19 infection using scoring methods such as the Brixia score, the RALE score, and the mCXR score. Both the clinical severity classification of the Vietnamese COVID-19 guideline and the qCSI had strong correlations to the three scores. In comparison to the Brixia score and the mCXR score, the RALE score was not only simpler to apply in clinical practice but also had stronger associations with SpO_2_ and the qCSI. Applying these imaging-based scoring systems in evaluating COVID-19 patients helps clinicians predict the prognosis to a certain extent and then make the right decision among numerous treatment choices. The RALE score and the qCSI are valuable tools for physicians in diagnosing and monitoring COVID-19 patients in the emergency department.
